# Local Ordering of Molten Salts at NiO Crystal Interfaces Promotes High‐Index Faceting

**DOI:** 10.1002/anie.202105018

**Published:** 2021-09-15

**Authors:** Raffaele Cheula, Mariano D. Susman, David H. West, Sivadinarayana Chinta, Jeffrey D. Rimer, Matteo Maestri

**Affiliations:** ^1^ Laboratory of Catalysis and Catalytic Processes Dipartimento di Energia Politecnico di Milano Via La Masa, 34 20156 Milano Italy; ^2^ Department of Chemical and Biomolecular Engineering University of Houston 4726 Calhoun Road Houston TX 77204-4004 USA; ^3^ SABIC Technology Center 1600 Industrial Blvd. Sugar Land Houston TX 77478 USA

**Keywords:** crystal habit, heterogeneous catalysts, high-index facet, nickel oxide, surface termination

## Abstract

Given the strong influence of surface structure on the reactivity of heterogeneous catalysts, understanding the mechanisms that control crystal morphology is an important component of designing catalytic materials with targeted shape and functionality. Herein, we employ density functional theory to examine the impact of growth media on NiO crystal faceting in line with experimental findings, showing that molten‐salt synthesis in alkali chlorides (KCl, LiCl, and NaCl) imposes shape selectivity on NiO particles. We find that the production of NiO octahedra is attributed to the dissociative adsorption of H_2_O, whereas the formation of trapezohedral particles is associated with the control of the growth kinetics exerted by ordered salt structures on high‐index facets. To our knowledge, this is the first observation that growth inhibition of metal‐oxide facets occurs by a localized ordering of molten salts at the crystal–solvent interface. These findings provide new molecular‐level insight on kinetics and thermodynamics of molten‐salt synthesis as a predictive route to shape‐engineer metal‐oxide crystals.

## Graphical abstract

With density functional theory, the phenomena that drive the shape selectivity of NiO particles crystallized by molten salt synthesis were studied. Trapezohedral NiO particles are shown to form in LiCl and KCl due to the interaction of locally ordered alkali‐chloride structures with the growing high‐index oxide facets, thus acting as capping agents. In the absence of salt, octahedral NiO formation is driven by the dissociative adsorption of water.

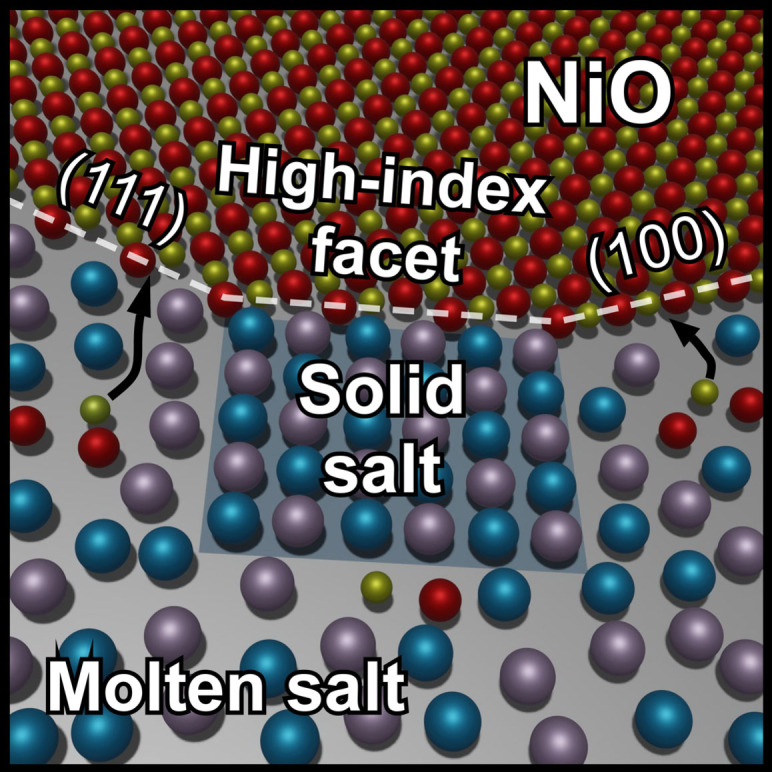

The catalytic activity of heterogeneous catalysts is strongly correlated to the composition and surface structure of the catalyst materials.[^1^, ^2^, ^3^, ^4^, ^5^, ^6^, ^7^] Several experimental and theoretical studies have reported that, for certain types of structure‐sensitive reactions, catalytic activity is enhanced by under‐coordinated active sites presented on high‐index (HI) crystal facets;^[4]^ therefore, the synthesis of catalysts exposing HI facets is particularly appealing for heterogeneous catalysis. Indeed, such materials could improve the efficiency and economics of catalytic processes. While several methodologies for the synthesis of materials exposing HI facets have been reported in the literature,[^8^, ^9^, ^10^] the fundamental understanding of the phenomena that control the final catalyst morphologies remains elusive. For metals, common strategies to control the catalyst final shape include the use of crystal growth modifiers, such as ambidentate solvents,^[10]^ and electrochemical square‐wave‐potentials.[^11^, ^12^] The final shape of the materials is sometimes associated with a lower surface free energy (*γ*_{*hkl*}_) of the desired {*hkl*} facets, induced by specific agents present in the growth environment. Alternatively, the particle morphology can be rationalized by evoking kinetic phenomena of crystal growth,^[13]^ often related to capping agents that selectively reduce the growth rate of particular crystal facets.[^14^, ^15^, ^16^] For metal oxides, few synthesis methods yielding HI particles have been reported.[^17^, ^18^, ^19^, ^20^, ^21^] One particularly effective strategy for the synthesis of HI metal oxides involves the use of non‐aqueous media, such as ionic liquids or by molten salt synthesis (MSS).^[22]^

High‐index nickel(II) oxide (NiO) particles are particularly interesting in heterogeneous catalysis, as NiO is a low‐cost industrial catalyst for oxidation reactions.[^19^, ^23^, ^24^, ^25^, ^26^] Susman et al.^[25]^ recently synthesized NiO particles by MSS in various salt media and observed that the final particle shape is strongly dependent on the growth environment. For syntheses in different alkali nitrates, they observed the production of cubes, octahedra, or cuboctahedra; however, a switch to media containing alkali chlorides directed the formation of HI facets. The synthesis comprised the calcination of Ni(NO_3_)_2_⋅6 H_2_O in an alkali salt at a heating rate of 2.5 °C min^−1^ to a maximum temperature of 550 °C. The samples were analyzed by scanning electron microscopy (SEM) after purification, showing significant differences in crystal shape. The MSS in KCl produced trapezohedra exposing NiO{311} HI facets; LiCl yielded {*h*11} facets where *h*≃5–6; and NaCl produced NiO particles with undefined shape. The thermal decomposition of Ni(NO_3_)_2_⋅6 H_2_O in the absence of salts was also studied. In air, NiO octahedra exposing {111} facets were formed, with complete NiO formation observed at ca. 350 °C. Similar experiments under a H_2_O‐free air flow, however, resulted in an irregular particle morphology. On this basis, it was postulated that the formation of octahedra is associated with the presence of H_2_O in the growth environment. Susman et al.^[25]^ also studied the NiO synthesis from NiCl_2_ in presence of KCl (and atmospheric air) to investigate if nitrates are necessary for {311} faceting. Their findings revealed that trapezohedra can crystallize in absence of nitrates, indicating that Cl^−^ and K^+^ ions are necessary for the formation of {311} HI facets. At the highest MSS calcination temperature (550 °C), nitrate mixtures are fully molten given their lower melting points (MPs), which are 255, 307, and 333 °C for LiNO_3_, NaNO_3_, and KNO_3_, respectively. In contrast, alkali chlorides are not fully molten as the MPs of LiCl, NaCl, and KCl (613, 801, and 771 °C, respectively) are well above the synthesis temperature; however, the presence Ni^2+^ and NO_3_^−^ species during crystallization can induce local eutectic mixtures with MP<550 °C where the liquid is in equilibrium with the non‐melted salts.

Herein, we employ density functional theory (DFT) within the generalized gradient approximation and Hubbard U corrections (GGA+U)[^27^, ^28^, ^29^] to study the interfaces between NiO and the different growth environments of MSS experiments with the aim of unraveling the chemistry behind the NiO shape selectivity achieved in different alkali chlorides. We use Quantum Espresso^[30]^ to perform the DFT calculations, with the Environ library[^31^, ^32^] for the implicit solvation models, and Grimme‐D3[^33^, ^34^] dispersion corrections. We focus on KCl and LiCl salts, which give rise to HI NiO particles, and NaCl, which produces irregular NiO shapes. For each alkali chloride, we optimize a solvation model that reproduces the experimental formation enthalpies of the salt in the solid and liquid phase. The crystal facets selected for the analysis resemble those observed experimentally, i.e., NiO{100}, {111}, {311}, and {511}. The latter is investigated in place of NiO{611} because it has similar morphology, but has higher symmetry. In our analysis, we investigate two scenarios in which HI facets are produced in the presence of capping agents: (1) via thermodynamic‐driven changes in particle shape, and (2) via kinetic hindering of the growth of specific crystal facets. The first scenario involves the attainment of the equilibrium shape wherein the adsorption of species available in the growth environment reduce the *γ*_{*hkl*}_ of certain {*hkl*} facets. This can result in different stable shapes at different experimental conditions, which can be represented using Wulff constructions.^[35]^ The second scenario consists of controlling the growth kinetics by capping agents that selectively bind to {*hkl*} surfaces and slow down (or enhance) the anisotropic rates of crystal growth. The final habit is comprised of facets characterized by the slowest growth rates (in the directions perpendicular to these facets) without reaching thermodynamic equilibrium.

We first investigated the thermodynamically stable structures of NiO in equilibrium with an oxidizing environment in the absence of any salt or water. Using ab initio thermodynamics,^[36]^ we calculated the *γ*_{*hkl*}_ of NiO crystal facets as a function of the chemical potential of O_2_ in the gas phase and, by considering an O_2_ partial pressure of 0.21 atm, as a function of temperature (Figure 1 a). Our calculations show that a stoichiometric NiO{100} termination is preferred in the entire temperature range tested. In agreement with previous theoretical and experimental studies,[^37^, ^38^, ^39^] we find that NiO{111} undergoes surface reconstruction, and it can potentially form two stoichiometric structures; however, the oxygen‐terminated interface (O) is more stable than the Ni‐terminated one (Ni). The facets NiO{311} and NiO{511} show over‐stoichiometric oxygen surface terminations (indicated as NiO{*hkl*}+O*) at temperatures below ca. 150 and 350 °C, respectively. At higher temperatures, they show stable stoichiometric surface terminations. The calculated most stable structures are presented in Figure S4. In the full range of temperatures under O_2_, NiO{100} is the facet with the lowest *γ*_{*hkl*}_, consistent with the Wulff construction prediction of a cube morphology in the absence of adsorbates. This agrees with gas‐phase syntheses of NiO, which result in cubic particles at high temperatures where adsorption of molecules from the environment is typically disfavored.^[40]^

**Figure 1:**
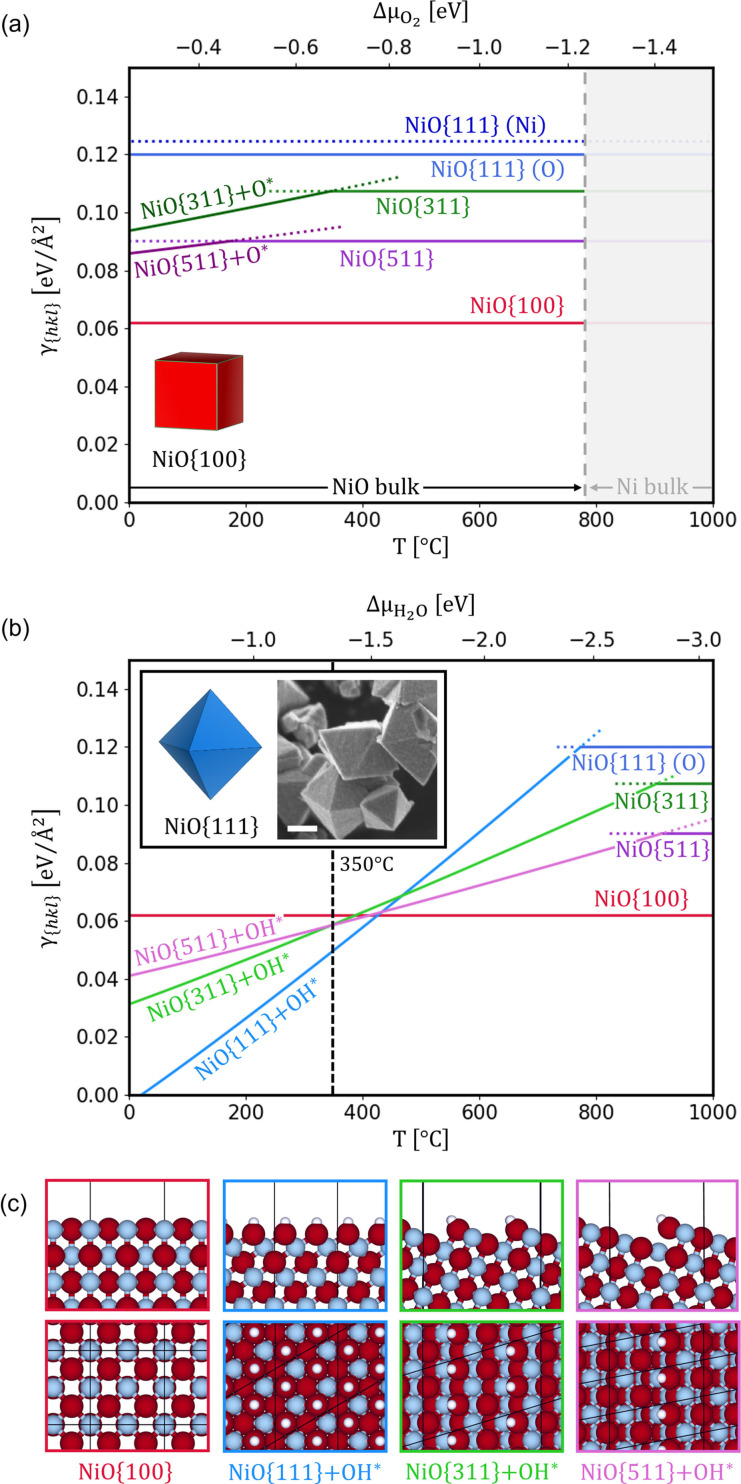
Specific surface free energies of NiO facets (a) as a function of O_2_ chemical potential and temperature (considering *p*(O_2_)= 0.21 atm), and (b) as a function of H_2_O chemical potential and temperature (considering *p*(H_2_O)=0.04 atm and *p*(O_2_)=0.21 atm). The grey area indicates the range of O_2_ chemical potential in which metallic Ni becomes the thermodynamically stable bulk phase. The calculated Wulff construction at 350 °C and (inset) corresponding octahedral crystals prepared in the presence of atmospheric air during a slow temperature ramp (Figure S2). Scale bar equals 400 nm. c) Calculated most stable NiO surface structures under atmospheric air at 350 °C (O in red, Ni in grey, H in white).

The NiO facet stability in the presence of H_2_O is investigated by analyzing hydroxylated surface structures, produced by H_2_O dissociation, which results in OH* species binding to Ni cations, and H* ions binding to O anions (and forming additional OH* species). In Figure 1 b, *γ*_{*hkl*}_ values are calculated in the presence of adsorbed OH* species as a function of temperature, considering a H_2_O partial pressure of 0.04 atm assuming saturated atmospheric (lab) air. The dissociative adsorption of H_2_O on NiO{100} is not favored at any temperature. Conversely, H_2_O dissociation is favored on NiO{111}, {311}, and {511} facets at temperatures lower than ca. 750, 890, and 900 °C, respectively. Below these temperatures, OH* adsorbates significantly reduce *γ*_{*hkl*}_ of corresponding facets due to their high surface concentration. These results are in good agreement with previous calculations of Marks and co‐workers^[37]^ who investigated the stability of OH* species on NiO{111} at different coverages with a meta‐GGA hybrid functional. The *γ*_{*hkl*}_ values were evaluated at conditions closer to experiment,^[25]^ i.e., ca. 350 °C, where octahedral NiO{111} faceting was observed (Figure 1 b). At this temperature, hydroxylated NiO{111}, {311}, and {511} are more stable than NiO{100}. In particular, *γ*_NiO{111}_ is significantly lower than the others considered, and the Wulff construction results in an octahedron; therefore, the formation of octahedra in air can be explained by the decrease of *γ*_{*hkl*}_ induced by the presence of adsorbed OH* species on NiO{111}. The optimized NiO surface structures at these conditions are represented in Figure 1 c.

A similar approach was used to study the structure of low‐ and high‐index NiO facets in the presence of alkali chlorides. In these systems, the cations (K^+^, Na^+^ or Li^+^) and anion (Cl^−^) can adsorb on the NiO facets and modify their energies and growth rates. In addition to studying the adsorption (and co‐adsorption) of salt ions at different coverages, we investigated the formation of solid salt structures at the salt‐NiO interface, showing different {*hkl*} surfaces in contact with the NiO facets. The formation of such solid‐like structures at the salt‐particle interface is a probable scenario, as the salts can be in solid–liquid equilibrium at the experimental conditions.^[25]^ Moreover, this phenomenon has already been observed in some theoretical studies on the specific heat of molten salts containing metal oxide nanoparticles.[^41^, ^42^] At this stage, we did not consider the presence of H_2_O or nitrates in the system, as experiments revealed that trapezohedra are produced under dry air and in the absence of nitrate ions.^[25]^ For charged adsorbates, the charge‐neutrality is maintained in the DFT calculations by adding counterions, which are stabilized by the solvation models (details in the Supporting Information).

Prior experiments reported the crystallization of NiO in the presence of alkali chlorides at 550 °C.^[25]^ Here, the results of ab initio thermodynamics do not explain the formation of HI facets as their *γ*_{*hkl*}_ are always higher than that of NiO{100}, either in the presence of KCl, LiCl, or NaCl (Figure S5). Interestingly, the alkali chloride structures that form on the NiO surfaces have the potential to operate as capping agents and tailor the shape selectivity of NiO particles by controlling their growth rates—an effect that is associated with the Gibbs binding energy (*G*_b_) of the capping agents.[^14^, ^15^, ^16^] In Figure 2 a, for each NiO stable clean surface termination (Figure 1 a), the *G*_b_ values of the structures representing the adsorption of K^+^ or Cl^−^ ions, the co‐adsorption of K^+^ and Cl^−^, and the formation of KCl solid structures in contact with the NiO facet are reported. For all the structures investigated, the same reservoir of K^+^ and Cl^−^ ions (i.e., the solid alkali salts at the experimental conditions) is considered in the calculation of *G*_b_. For each condition, we show the most stable structures for each potential NiO surface termination (Figure 2 a, top row). The adsorption of K^+^ is favored on NiO{100} and NiO{311}+O*, whereas on NiO{111} and NiO{511} terminations the co‐adsorption of K^+^ and Cl^−^ is preferred. NiO{311} is the only facet on which local ordering of crystalline KCl is favored. The resulting solid‐solid structure at the interface between KCl and NiO{311} shows a very strong *G*_b_, thus densely ordered KCl at the crystal interface can act as a capping agent hindering NiO growth in the [311] direction. Moreover, when we compare the *G*_b_ on different NiO terminations, we do not find any apparent correlation between the adsorption of K^+^ or Cl^−^ ions and the exposure of NiO{311} facets in the final particles. Instead, the *G*_b_ between crystalline KCl structures and NiO facets indicated a much stronger interaction with NiO{311} compared to other facets. As a result, we associate the shape selectivity of KCl (Figure 2 b) to the localized ordering of crystalline KCl at the NiO{311} surface, forming a very stable solid‐solid interface, and hindering the growth of that specific facet. It should be noted that this solid‐solid KCl‐NiO interface can still be dynamic, as upon NiO crystallization there is a release of KCl from the conversion of the KNiCl_3_ intermediate,^[25]^ which provides further mobility to the KCl phase to adapt to the evolving NiO surface.

**Figure 2:**
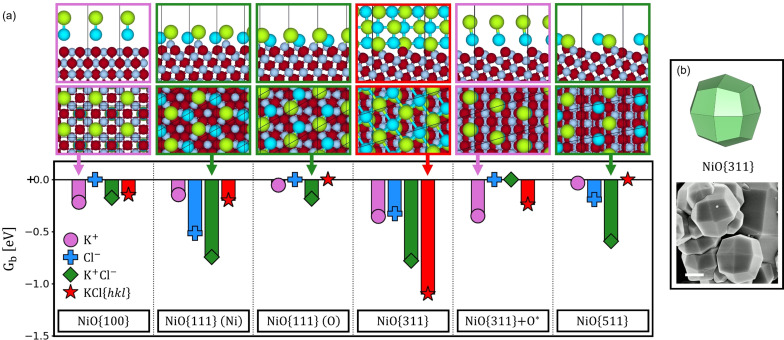
a) (*Bottom*) Gibbs binding energies (per unit cell) of NiO surface structures involving the adsorption of K^+^, Cl^−^, coadsorbed K^+^ and Cl^−^ ions, and crystal KCl{*hkl*} salt structures on various NiO surface terminations (indicated), calculated at 550 °C and with respect to clean NiO surfaces. (*Top*) Lateral and top views of the most strongly bound agents for each NiO termination; the counterions, needed for charge‐neutrality (and stabilized by the implicit solvent), are also shown (O in red, Ni in grey, K in cyan, Cl in green). b) Model exposing {311} facets and experimental NiO particles produced by MSS in KCl. Scale‐bar equals 1 μm.

Similar *G*_b_ values were calculated for the LiCl system (Figure 3 a) where it was observed that Li^+^ adsorption is preferred on NiO{311}+O*, Li^+^ and Cl^−^ co‐adsorption is favored on all remaining facets except for NiO{511}, which favors the crystallization of LiCl. When the adsorption of the salt ions are analyzed, there is no appreciable correlation between *G*_b_ and the experimental particle shape; however, for the LiCl crystal‐NiO interface, the adsorption is instead stronger for NiO{511} compared to the other facets. As a result, we associate the shape selectivity of LiCl to the crystallization of LiCl on NiO{511} facets. A model particle exposing {511} facets as well as an SEM image of particles obtained experimentally are represented in Figure 3 b.

**Figure 3:**
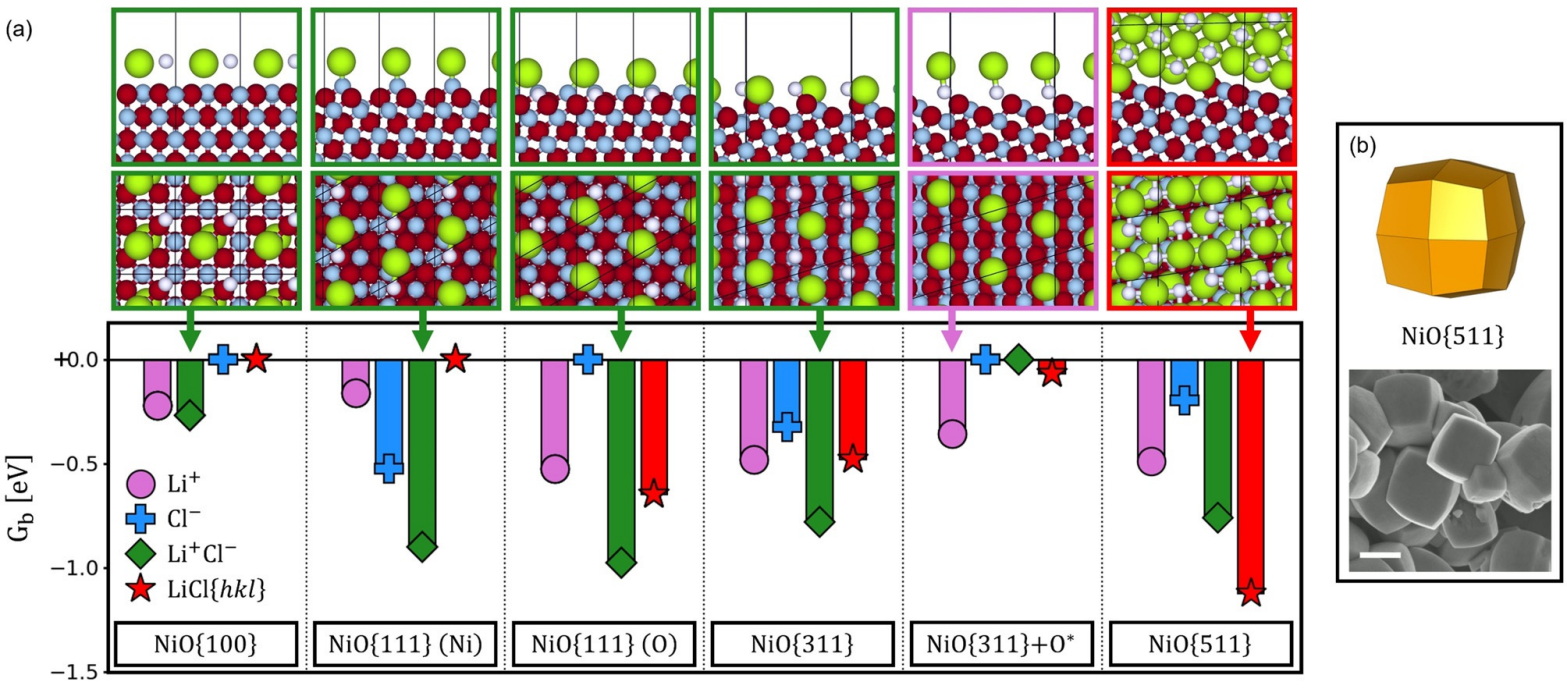
a) (*Bottom*) Gibbs binding energies (per unit cell) of NiO surface structures involving the adsorption of Li^+^, Cl^−^, coadsorbed Li^+^ and Cl^−^ ions, and crystal LiCl{*hkl*} salt structures on various NiO surface terminations (indicated), calculated at 550 °C and with respect to clean NiO surfaces. (*Top*) Lateral and top views of the most strongly bound agents for each NiO termination; the counterions, needed for charge neutrality (and stabilized by the implicit solvent), are also shown (O in red, Ni in grey, Li in white, Cl in green). b) Model exposing {511} facets and experimental NiO particles produced by MSS in LiCl. Scale‐bar equals 1 μm.

For the case of NiO synthesis in NaCl, which yields particles with undefined shape,^[25]^ we calculated that the formation of locally ordered structures is not favored on any of the investigated {*hkl*} facets. This confirms that the local ordering of the alkali chloride is needed for the formation of HI NiO particles. The results for NaCl media are presented in Figure S6.

A schematic representation of the proposed mechanism of alkali chloride induced shape selectivity of NiO particles is shown in Figure 4, using the KCl system as an example. On NiO{100} and {111} facets, the liquid‐like K^+^ and Cl^−^ media do not fully cover the surfaces; and the dynamic adsorption and desorption of ions allows for growth units to reach NiO surfaces and promote crystal growth along the [100] and [111] directions. However, on NiO{311} surfaces, an ordered solid‐like KCl structure preferentially forms at the crystal‐salt interface owing to its high *G*_b_. This local ordered salt structure significantly reduces the probability that growth units would reach the surface, hindering NiO crystal growth in the [311] direction. As a result, the final shape of the particle will display {311} facets, which are protected by the adsorbed KCl ordered structure, even if its *γ*_{*hkl*}_ is higher than the low‐index facets.

**Figure 4:**
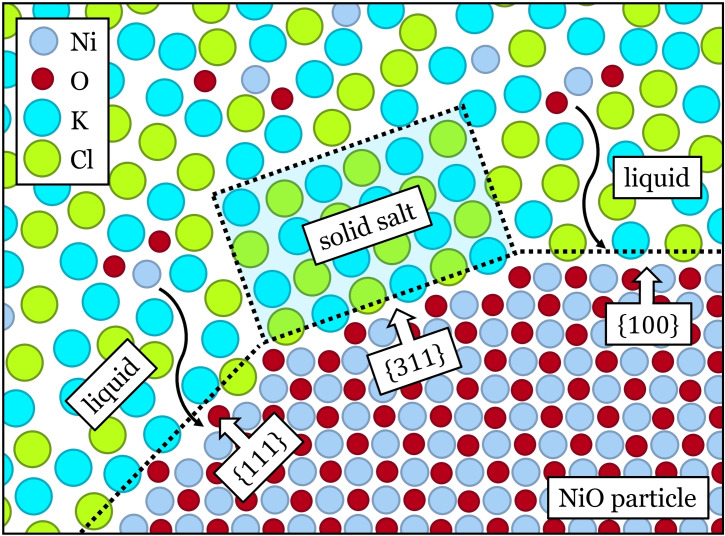
Schematic representation of the selective growth of NiO facets due to the adsorption of crystalline alkali chloride domains (depicted here for KCl) onto NiO {311} facets. The ordered crystalline KCl multilayer hinders growth in the [311] direction compared to facets exposed to liquid‐like KCl that grow.

In summary, we investigated the use of MSS media to tailor NiO crystal shape under different experimental conditions involving alkali chloride salts that produce HI facets. Two different scenarios were investigated: the change of thermodynamically stable particle shape induced by the presence of *γ*_{*hkl*}_ modifiers, and the control of the crystal growth provoked by the adsorption of capping agents. We showed that the formation of octahedral particles is driven by the adsorption of water molecules that form OH* groups and stabilize the NiO{111} facet. To interpret NiO faceting induced by the judicious selection of alkali chlorides, we analyzed the adsorption of alkali metal salt ions and counterion (K^+^, Li^+^, Na^+^, and Cl^−^) as well as the induced ordering of ions in local proximity to crystal surfaces, thus forming solid‐solid interfaces between NiO HI facets and alkali salts. This allowed identifying the role of the local ordering of the alkali chlorides at the NiO surfaces, which is a plausible scenario considering the salts are in solid–liquid equilibrium at experimentally relevant temperatures. The formation of NiO{311} HI facets during MSS in KCl is associated with the formation of an interface involving crystalline KCl that selectively adsorbs on {311} surfaces. The formation of NiO{511} facets, observed using LiCl, is associated with the crystallization of the LiCl salt on {511} surfaces. Collectively, these results explain our reported experimental observations, thus providing a starting point for the fundamental understanding of shape control in MSS of metal oxide particle crystallization. Habit modification by altered *γ*_{*hkl*}_ of crystal facets (or Wulff constructions) is a widely accepted explanation of crystal shape engineering; however, few studies provide a molecular‐level description of the mechanisms and phenomena responsible for altering the thermodynamics of crystal‐solvent interfaces. Here, we show for the first time that a locally ordered salt in a molten salt medium can behave as a capping agent regulating the anisotropic growth rates of a metal oxide. The development of theoretical models, such as those presented here, has great potential in heterogeneous catalysis, as models can guide the synthesis of new materials with desired shapes and targeted functionalities.

## Conflict of interest

The authors declare no conflict of interest.

## Supporting information

As a service to our authors and readers, this journal provides supporting information supplied by the authors. Such materials are peer reviewed and may be re‐organized for online delivery, but are not copy‐edited or typeset. Technical support issues arising from supporting information (other than missing files) should be addressed to the authors.

Supporting Information
