# Supplementary material for: Local Ordering of Molten Salts at NiO Crystal Interfaces Promotes High‐Index Faceting
Source: Angew Chem Int Ed Engl. 2021 Sep 15;60(48):25391–6. doi: 10.1002/anie.202105018 (PMC9290742; doi:10.1002/anie.202105018)
Supplement: Supplementary file 1 — Supporting Information [file ANIE-60-25391-s001.pdf]

Angewandte Chemie
Eine Zeitschrift der Gesellschaft Deutscher Chemiker

Supporting Information

Local Ordering of Molten Salts at NiO Crystal Interfaces Promotes High-Index Faceting

Raffaele Cheula, Mariano D. Susman, David H. West, Sivadinarayana Chinta, Jeffrey D. Rimer, and Matteo Maestri**

anie_202105018_sm_miscellaneous_information.pdf

Table of Contents

1. Experimental results (page 2)
2. Methods (page 3)
3. Results and Discussion (page 4)
4. References (page 6)

1. Experimental results

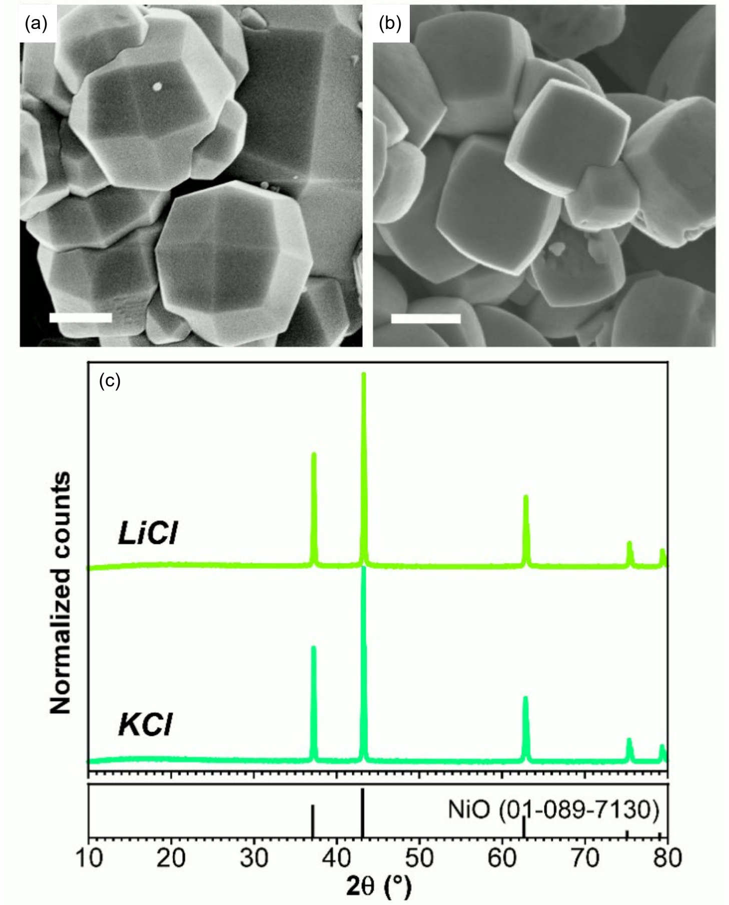

Figure S1. (a,b) Scanning electron microscopy (SEM) images of NiO particles produced by molten salt synthesis in (a) KCl and (b) LiCl, using $Ni(NO_3)_2{\cdot}6H_2O$ as oxide precursor (550 °C for 1 h). Scale bars equal 1 µm. (c) Corresponding powder X-Ray diffraction patterns. The ICDD NiO XRD reference is indicated below.

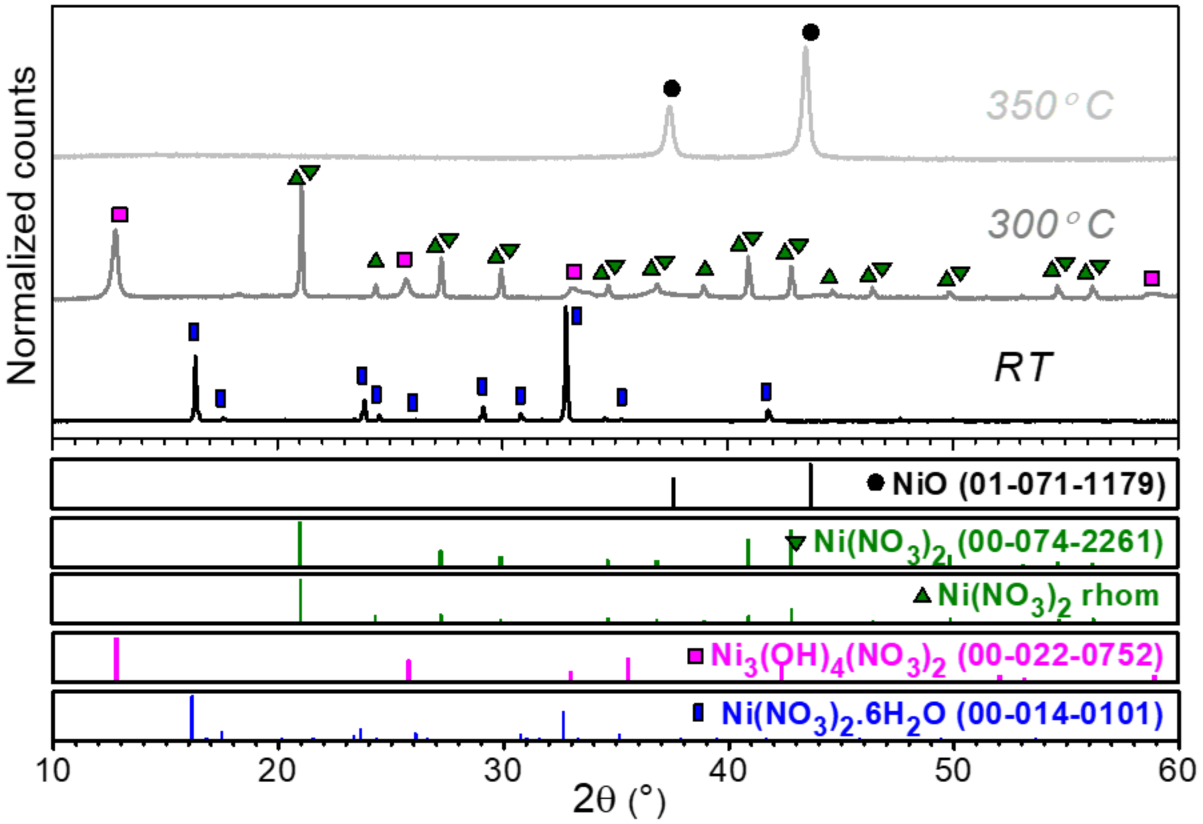

Figure S2. Powder X-ray diffraction patterns of $Ni(NO_3)_2{\cdot}6H_2O$ decomposed at a heating rate of 2.5 °C/min in air (temperatures indicated). Reference XRD patterns from ICDD or, for rhombohedral $Ni(NO_3)_2$, calculated from single crystalline diffraction data (Giester et al., *Z. Kristallogr.* **2008**, 223, 408-417) using the software Diamond 3.2k.

2. Methods

2.1 Electronic structures calculations

Electronic-structure density functional theory (DFT) calculations are performed using the Quantum Espresso[1] suite of codes, with GGA-PBE[2] ultrasoft pseudopotentials and a plane wave basis set. The plane wave and electronic density cut-off energies are set to 50 Ry and 400 Ry, respectively. Spin-polarized calculations are employed, due to the anti-ferromagnetic behavior of NiO. A Monkhorst-Pack of 4×4×4 *k*-points sampling is used for the unit cell of bulk NiO (a 2×2×2 rock salt unit cell, with spins alternated in the [111] direction).[3] A proportional grid is used to map the Brillouin zone for other bulk phases, surface slabs, and interface slabs. The method of DFT+U[4,5] is employed to represent the strongly correlated electrons of NiO. The U correction is applied to the *d* orbitals of Ni atomic species, using a value of U (7.8 eV) obtained with density functional perturbation theory (DFPT), as proposed by Cococcioni and co-workers.[3,6] The Grimme-D3[7,8] correction is employed in all the calculations to represent long range interactions, particularly important for ionic salts. Periodic supercells with inversion center and a slab height thicker than 8 Å are employed to represent the NiO surfaces. A vacuum of 12 Å in the z direction is introduced between periodic slabs. All the atoms in the supercells are allowed to relax. A cubic cell with a side length of 12 Å is chosen for gas phase calculations. For atomic position optimizations (relax calculations), the convergence force threshold selected is 10^{-3} Ry/Bohr, and a difference in energy lower than 10^{-4} Ry between two consecutive self-consistent field (SCF) steps is required for convergence. Vibration analyses are performed with the finite-differences method, as implemented in the Atomic Simulation Environment (ASE) library,[9] from the diagonalization of the Hessian matrix, obtained applying displacements of 0.01 Å in the three cartesian directions to the selected atoms. Normal frequencies are evaluated for the atoms of the adsorbed species (if present) and for the atoms of the first layer of the surface structures under investigation. For salt-NiO solid-solid interfaces, the vibrations are calculated for the atoms of the two adjacent layers of the solids (one salt layer and one NiO layer).

For bulk NiO, the vibrational free energy is obtained from finite-differences phonons calculations (with a 2×2×2 supercell), as implemented in the ASE library.[9] For interface structures, phonons calculations are prohibitive due to the large sizes of the supercells. Therefore, we evaluate only the frequencies at Gamma-point and we neglect the in-plane periodicity of the structures (diagonalization of the Hessian matrix). To validate these approximations, we calculate vibrational free energy and zero-point energy (ZPE) of a sample structure (i.e., the interface between KCl{100} and NiO{311}) with a phonons calculation and we compare the results with the ones obtained with the methodology employed in this work. In the phonons calculation, we evaluate vibrational modes for the same atoms of the original calculation (one salt layer and one NiO layer), and we use a 2×2×1 supercell (the results are relative to the original supercell). The results obtained with the methodology employed in this work (diagonalization of the Hessian matrix) are a ZPE of 0.773 eV and a vibrational Helmoltz free energy (at 550 °C) of –4.075 eV. With the phonons calculation, we obtain a ZPE of 0.723 eV and a vibrational Helmoltz free energy (at 550 °C) of –4.201 eV. The results obtained with the two methods are in reasonably good agreement; therefore, the approximations introduced do not affect our main conclusions.

2.2 Solvation model

The presence of liquid alkali chlorides is accounted for with the implicit solvent method, as implemented in the Environ library.[10,11] The main parameters of the model are calculated with a Bayesian 3D optimization to reproduce the experimental formation enthalpy of solid and liquid alkali chlorides at 550 °C. A similar approach was used by Andreussi et al.[10], who produced a solvation model for several molecules in water from the optimization of the parameters of the implicit solvent model. The soft-sphere continuum solvation (SSCS) model[11] is employed, which uses a smooth 3D solvent interface defined as a function of the interlocking smooth spheres centered on the positions of the nuclei of the system. The solvation radii (distance between the solvent interface and the nuclei) are defined for each atomic species and they are scaled with a global parameter, α_s. The numerical spread of the soft-sphere functions is set to 0.2, and a value of $\alpha_s = \sqrt{2}$ is employed to ensure that the continuum solvent is not defined inside the cubic bulk structures of the alkali chlorides. The dielectric constant of the liquid alkali chlorides (ε_{KCl}, ε_{LiCl}, and ε_{NaCl}) and the solvation radii (r_i) of cations (K^+, Li^+, or Na^+) and anions (Cl^-) are obtained with a Bayesian 3D optimization procedure based on the scikit-learn Python library.[12,13] The procedure is designed to minimize the errors in the formation energies (at 550 °C) of cations and anions with different coordination number (CN) contained in a set of selected alkali salt structures. The set comprises the separated salt ions, a salt monomer, the {100}, {110}, and {111} salt surfaces. The function to minimize (for KCl) is defined as:

$$\mathbf{fun}(\boldsymbol{\varepsilon}_{\mathbf{KCl}}, \mathbf{r}_{\mathbf{K^+}}, \mathbf{r}_{\mathbf{Cl^-}}) = \sum_{\mathbf{i}} \left| \mathbf{E}_{\mathbf{f,i}}^{\mathbf{DFT}}(\mathbf{CN_i}) - \mathbf{E}_{\mathbf{f,i}}^{\mathbf{target}}(\mathbf{CN_i}) \right| \quad \text{(S1)}$$

where $E_{f,i}^{DFT}$ is the formation energy of the atoms with CN lower than 6, calculated using the implicit solvent model with the parameters ε_{KCl}, r_{K^+}, and r_{Cl^-}; $E_{f,i}^{target}$ is the target value for the formation energy, which is calculated from the experimental formation energies of solid (E_f^{solid}) and liquid (E_f^{liquid}) alkali chlorides at 550 °C. The values of experimental formation energies are obtained from NIST-Janaf thermochemical tables,[14] where E_f^{liquid} is calculated adding to E_f^{solid} the value of enthalpy of fusion of the salt. In the calculations, the interatomic distances are fixed to the values of a bulk structure with a formation energy equal to E_f^{solid}. The target values, E_f^{target}, are calculated as weighted average between E_f^{solid} and E_f^{liquid}, with the following formula (6 is the maximum CN in bulk rock salt structures):

$$\mathbf{E_f^{target}(CN) = \frac{CN}{6} E_f^{solid} + \frac{(6 - CN)}{6} E_f^{liquid}} \quad \text{(S2)}$$

With Equation S2, we set E_f^{target} equal to E_f^{solid} for the bulk alkali salt, and E_f^{target} equal to E_f^{liquid} for the separated alkali salt ions surrounded by the implicit solvent representing the liquid phase. The starting values for the solvation radii are set to the ionic radii obtained with Bader charges analysis of the alkali chlorides bulk structures. In Table S1 are reported the E_f^{DFT} calculated with the optimized implicit solvent models of liquid KCl, LiCl, and NaCl, and the comparison with the corresponding target values. For the LiCl system, the optimized parameters resulted in values of ε_{LiCl} = 74, r_{Cl^-} = 1.62 Å, and r_{Li^+} = 1.04 Å. For the KCl system we obtained: ε_{KCl} = 55, r_{Cl^-} = 1.61 Å and r_{K^+} = 1.38 Å. For the NaCl system we obtained: ε_{NaCl} = 47, r_{Cl^-} = 1.61 Å and r_{Na^+} = 1.14 Å. The dielectric constants obtained from the optimization are relatively high if compared to the ones of the bulk alkali chlorides, and this in part reflects the higher ionic and dipolar contribution to the polarization of liquid substances, and in part is due to the empirical fitting of the parameters of the model. Moreover, a high dielectric constant reduces the unphysical lateral interactions between the ions in the liquid phase, which are very strong in vacuum DFT calculations. The parameters of the implicit solvent models representing KCl, LiCl, and NaCl are obtained for the systems at 550 °C; however, they can be easily adjusted to reproduce systems at different temperatures with a small set of cheap DFT calculations. We expect that the error that we would introduce by applying the model at a temperature, T, different than 550 °C, has the same order of magnitude of the difference in the enthalpy of the liquid alkali chlorides calculated at T and at 550 °C. From NIST-Janaf tables[14], we estimated that a temperature difference of 100 °C corresponds to an enthalpy difference of 0.076 eV for KCl, 0.065 eV for LiCl, and 0.065 eV for NaCl. Such values can be used to estimate the errors in using the model at T different than 550 °C.

Table S1. Formation energies of the alkali chlorides atoms with different coordination numbers (CN) in the structures used to fit the implicit solvent model. E_f^{target} are the target values of formation energy obtained from thermochemical tables, E_f^{DFT} are the values calculated with DFT and the optimized implicit solvent model.

**KCl**				
	CN [−]	E_f^{target} [eV/KCl]	E_f^{DFT} [eV/KCl]	error [eV/KCl]
K^+ Cl^- ions	0	0.700	0.680	-0.020
KCl monomer	1	0.655	0.660	0.005
KCl {110}	3	0.564	0.629	0.065
KCl {111}	4	0.519	0.507	-0.012
KCl {100}	5	0.473	0.471	-0.002
KCl solid	6	0.428	0.430	0.002

**LiCl**				
	CN [−]	E_f^{target} [eV/LiCl]	E_f^{DFT} [eV/LiCl]	error [eV/LiCl]
Li^+ Cl^- ions	0	0.605	0.594	-0.011
LiCl monomer	1	0.571	0.590	0.019
LiCl {110}	3	0.502	0.541	0.039
LiCl {111}	4	0.468	0.461	-0.007
LiCl {100}	5	0.434	0.448	0.014
KCl solid	6	0.399	0.400	0.001

**NaCl**				
	CN [−]	E_f^{target} [eV/NaCl]	E_f^{DFT} [eV/NaCl]	error [eV/NaCl]
Na^+ Cl^- ions	0	0.708	0.682	-0.026
NaCl monomer	1	0.659	0.622	-0.037
NaCl {110}	3	0.562	0.641	0.080
NaCl {111}	4	0.513	0.488	-0.025
NaCl {100}	5	0.464	0.456	-0.009
NaCl solid	6	0.416	0.418	0.002

2.3 Interface structures

The interfaces between the crystal facets of NiO and the solid alkali chlorides are represented with sandwich-like supercells, produced with a methodology that preserves the periodicity of the two structures in contact and minimizes their internal stress. The procedure consists of the steps schematized in Figure S3. In the first step, the two structures are reduced to their primitive cells, and the base vectors of their unit cell are obtained (Figure S3a). We call here v_1 and v_2 the base vectors of the NiO surface, w_1 and w_2 the base vectors of the alkali salt structure. In the second step, new structures are obtained from the original ones, by repeating the surface structures in their periodic directions and cutting them with new surface vectors (Figure S3b). The new vectors (V_1, V_2, W_1 and W_2) are obtained as linear combination of the original ones (v_1, v_2, w_1, and w_2), with the formulas reported in Figure S3, where a_{ij} are the elements of a matrix of integers. Those integers change iteratively each from 1 to a maximum defined to impose a maximum in the surface area of the resulting sandwich-like supercell. For each iteration,

the base vectors of the two structures are compared, and if they are compatible, the structure is selected as a possible candidate for the calculation. The compatibility is confirmed if the following three criteria are satisfied: the difference between $|V_1|$ and $|W_1|$, divided by $|V_1|$ is below a threshold value (t_{len}), the difference between $|V_2|$ and $|W_2|$, divided by $|V_2|$ is below t_{len}, and difference between the angle α (between V_1 and V_2) and the angle β (between W_1 and W_2) is below another threshold value (t_{angle}). In our calculations, t_{len} is set to 10%, and t_{angle} is set to 20°. However, these thresholds are needed only to screen the possible candidates of the calculations. For each candidate, the structure of the salt surface is rotated and stretched to match the dimensions of the NiO (Figure S3c). In this way, we guarantee continuity to the periodic boundary conditions of the DFT calculations. The stress in the alkali salt is then estimated from the stiffness tensor of the bulk and the deformation matrix. Among the possible candidates, the best structures worth the DFT investigation (usually 1 or 2 structures) are chosen by comparing the calculated stress in the alkali salt cells and by visual inspection, preferring the structures that show a match between the position of the alkali salt ions and the adsorption sites on the NiO. For each chosen structure, the sandwich-like supercell representing the interface is created by positioning the alkali salt structure on both sides of the NiO structure (Figure S3d). Then, the z vector of the final supercell is adjusted to guarantee periodicity also in the z direction, and variable-cell DFT calculations (i.e., calculations in which length and direction of the supercell vectors are allowed to change in the energy minimization procedure) are employed to minimize the strain induced by the stretching of the structures.

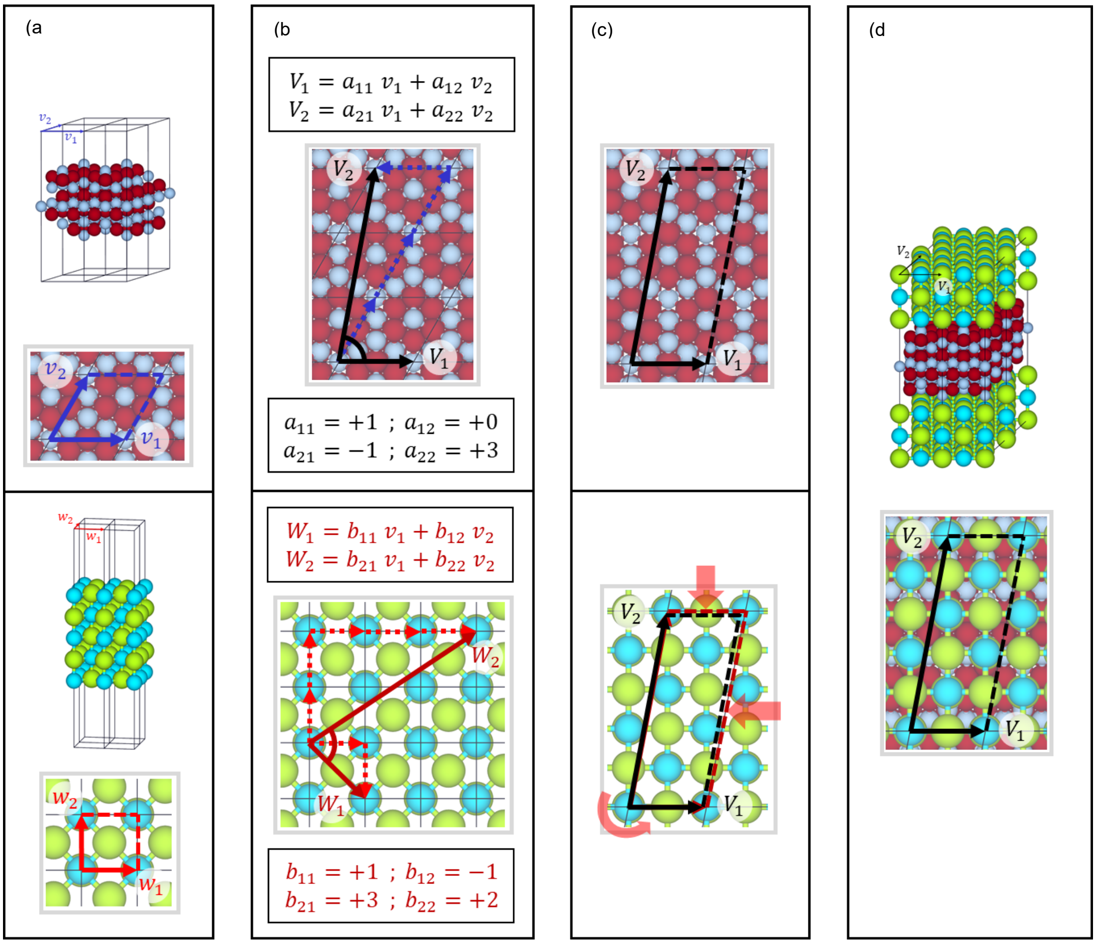

Figure S3. Schematic procedure for the creation of sandwich-like supercells representing the interface between NiO facets and alkali salt structures. As an example, it is reported the step-by-step modelling of the interface between NiO{111} (Ni) and KCl(100) (O in red, Ni in grey, K in cyan, Cl in green).

Owing to their elastic properties, the strain is introduced in the alkali salt structures. In our calculations, the height of alkali salt structures (ca. 8-10 Å) is selected to guarantee the formation of a bulk-like solid alkali salt region in the simulation cell without introducing a large amount of strain in the supercell. For example, for the structure representing the interface between KCl{100} and NiO{311}, we calculated that the strain energy of the stretched KCl{100} structure is 0.17 eV per NiO{311} unit cell, which corresponds to 5.5 meV/Å^2. We calculated such a value of strain energy as follows: we removed the atoms of

NiO{311} from the interface supercell; we removed the vacuum introduced, obtaining a deformed bulk KCl structure; we calculated the energy of the structure, and we subtracted the energy of the same number of atoms in the bulk KCl. As the strain energy is proportional to the volume, its effect is concomitantly more pronounced with increasing thickness of the KCl structure. In particular, for the structure considered (in which the KCl{100} structure is 9 Å thick), it increases 5.5 meV/Å^2 every 9 Å. Above a certain critical thickness, the internal strain will either induce the formation of dislocations or the formation of an interface with the liquid phase (when the KCl{100} growth becomes unfavorable). In our calculations, we did not investigated structures with different thicknesses, but we imposed the same thickness to all interface structures (8-10 Å). With the procedure described above, we calculated the strain energies of all the stretched alkali salts in the interface structures, obtaining values comprised between 0.1 and 0.3 eV. As a result, the effect of the strain energy is similar for the different structures, and it is small compared to the differences in the Gibbs binding energies of interface structures (0.5-1.0 eV per unit cell, red bars in Figure 2 and 3). Therefore, our main conclusions and in particular the correlation between the Gibbs binding energy of interface structures and the experimental particle shape are maintained despite that, on different NiO structures, the alkali salts could form structures with different heights and produce dislocations.

2.4 First principles thermodynamics

The specific surface free energies ($\gamma_{\{hkl\}}$) of the {*hkl*} facets of NiO in a chemical environment are obtained with the method of *ab initio* thermodynamics,[15]

$$\boldsymbol{\gamma}_{\{hkl\}} = \frac{\mathbf{1}}{\mathbf{S_A}}\left(\mathbf{G_{slab}} - \mathbf{N_{NiO}g_{bulk}} - \sum\nolimits_{i}\mathbf{N_i\mu_i}\right) \quad \text{(S3)}$$

where G_{slab} is the Gibbs free energy of the slab structure, containing N_{NiO} monomers of NiO, and N_i additional adsorbed species. g_{bulk} is the Gibbs free energy of one monomer in the bulk NiO, S_A is the surface area of the slab, equal to the double of the area of the symmetric simulation cell. μ_i are the chemical potentials of the species in the reservoirs of the system (i.e., O_2 and H_2O in the gas phase, KCl, LiCl, and NaCl in their solid bulk phase). Those chemical potentials are calculated at the experimental conditions (temperature, concentration), from NIST-Janaf thermochemical tables,[14] with the following formula:

$$\boldsymbol{\mu}_\mathbf{i} = \mathbf{E_i} + \mathbf{E_i^{ZPE}} + \Delta\boldsymbol{\mu}_\mathbf{i}^\mathbf{0} + \mathbf{k_B T \ln(P_i/P^0)} \quad \text{(S4)}$$

where E_i is the electronic energy of a molecule at 0 K, and E_i^{ZPE} is the zero-point energy. $\Delta\mu_i^0$ is the chemical potential calculated with respect to the reference at 0 K, obtained from tabulated values of enthalpies and entropies (at the pressure P^0). k_B is the Boltzmann constant, T is the temperature, and P_i is the partial pressure. The pressure term is considered only for gas-phase species and it is obtained within the ideal gas approximation. At the experimental reaction conditions (550 °C, 1 atm), the values of $\Delta\mu_i^0$ obtained from NIST-Janaf tables are: −1.78 for O_2, −1.64 for H_2O, −0.77 for KCl, −0.57 for LiCl, −0.68 for NaCl.

For the structures involving the adsorption of cations or anions, the counterions are added also in the simulation cell, in order to maintain charge-neutrality in the DFT periodic calculation and hinder unphysical coulomb interactions between periodic replicas of the simulation cell. The introduced counterions are stabilized by the solvation models, which simulate the presence of the liquid alkali chlorides and reproduce the experimental formation energies of the alkali chlorides at the temperature under investigation (550 °C).

The Gibbs free energy of solid systems is evaluated from electronic energy DFT calculations and vibrational analyses. For clean NiO surfaces, in the presence of O* and OH* adsorbates, and for the case of salt-NiO solid-solid interfaces, the harmonic limit model is applied, as implemented in the atomic simulation environment (ASE) library,[9] neglecting the pressure and concentration terms.[15] For adsorbed alkali chloride monomers, the entropy is calculated as a 2/3 of the entropy of liquid alkali chlorides (extrapolated to 550 °C) plus a 1/3 of the entropy of the solid alkali chlorides at 550 °C. As a result, we model the adsorbed alkali chlorides as 2D liquids. This is because of the high mobility of alkali salt ions on NiO surfaces and to achieve conservative results.

Gibbs binding energies ($G_{b,i}$) are calculated to quantify the adsorption strength of the agents that can adsorb on the NiO crystal facets, and therefore to identify the configuration of atoms at the surface that is thermodynamically most favored,

$$\mathbf{G_b} = \frac{\mathbf{1}}{\mathbf{N_{UC}}}\left(\mathbf{G_{slab}} - \mathbf{N_{UC}G_{slab}^{clean}} - \sum\nolimits_{i}\mathbf{N_i\mu_i}\right) \quad \text{(S5)}$$

where G_{slab} is the Gibbs free energy of the slab with the adsorbates, G_{slab}^{clean} is the Gibbs free energy of the clean slab, and N_{UC} is the number of unit cells corresponding to the slab with the adsorbates. In the comparison of the stability of the investigated structures, we compare the Gibbs free energies per unit cell. In this way, we can compare the stability of structures with different periodicities and the adsorption of molecules at different coverage.

To make a fair comparison between the thermodynamic stability of different structures, the Gibbs binding energies are calculated from the same reservoir state. Moreover, such reservoir states must be present at the experimental conditions. In our calculations for each different alkali chloride, the same reservoir state is set for all the systems considered (involving adsorption, co-adsorption and interfaces). Indeed, the chemical potential (μ_i) of the solid alkali salt at the experimental conditions (550 °C) is used in Equation S5, given that solid alkali salts are present during crystal growth, as they are in solid-liquid equilibrium. Those values of chemical potential are calculated with Equation S4 using the NIST-Janaf thermochemical tables.

In a first approximation, we can associate the hindering effects to the Gibbs binding energy of the capping agents ($G_{b,A^*}^{\{hkl\}}$). Indeed, the growth rates of the NiO crystal facets ($r^{\{hkl\}}$) is proportional to the free sites ($\theta_*^{\{hkl\}}$) available for the adsorption of the growth units:

$$\mathbf{r}^{\{hkl\}} \propto \boldsymbol{\theta}_*^{\{hkl\}} = \mathbf{1} - \boldsymbol{\theta}_{\mathbf{A}*}^{\{hkl\}} \tag{S6}$$

The ratio between the coverages of the capping agents (θ_{A*}^{hkl}) and free sites (θ_*^{hkl}) can be evaluated from the Gibbs binding energy of the capping agents ($G_{b,A^*}^{\{hkl\}}$):

$$\frac{\boldsymbol{\theta}_{\mathbf{A}*}^{\{hkl\}}}{\boldsymbol{\theta}_*^{\{hkl\}}} \propto \mathbf{exp}\left(-\frac{\mathbf{G}_{\mathbf{b,A^*}}^{\{hkl\}}}{\mathbf{k_B T}}\right) \tag{S7}$$

The adsorption of capping agents can also inhibit the attachment of growth units by steric hindrance, and this effect enhances the one of the blocking of the free sites. As a result, if the adsorption of a capping agent is much stronger on a certain NiO facet with respect to the others, we can identify that agent as the growth modifier responsible for the final shape of the particle exposing that facet.

3. Results and Discussion

The Gibbs binding energy ($\mathrm{G_b}$) and the corresponding surface free energy ($\gamma_{\{hkl\}}$) of the structures investigated, at the experimental conditions (550 °C and 1 atm), are reported in Table S2 (KCl), Table S3 (LiCl), and Table S4 (NaCl). The set comprehends structures describing the adsorption (and co-adsorption) of cations and anions from the alkali chlorides at different coverage, and formation of extensive salt structures on top of the NiO facets. The coverage reported in the table is defined as ratio between the number of adsorbed species and the number of equal adsorption sites on the NiO facet, and it is reported in monolayer (ML) units. The solid structures are denoted with the {*hkl*} Miller index that they show in contact with the NiO facets.

The NiO surface terminations under investigation were selected by choosing the stable clean NiO surface structures between 0 and 1000 °C (Figure 1a). Those structures are represented in Figure S4, and comprise stoichiometric structures (i.e., NiO{100}, NiO{111} (O), NiO{111} (Ni), NiO{311}, NiO{511}), as well as over-stoichiometric structures (i.e., NiO{311}+O* and NiO{511}+O*). Moreover, we also investigated NiO{111}+O*, despite its high surface free energy. The investigated over-stoichiometric structures have an overall O* coverage of 1.00 ML (indicated as +0.50ML in Tables S2, S3, and S4, because the O* coverage of stoichiometric structures is already 0.50 ML). For each NiO surface termination, interfaces with the most stable alkali salt facets (i.e., {100}, {111}, {110}) were usually considered. For the stepped NiO{511}, instead, the interfaces with the stepped {310} and {311} alkali surfaces were considered, because their geometrical structures matched. The best interface structures were obtained with the methodology described in Section 2.3.

In Figure S6, for each stable clean surface termination of NiO (Figure 1a and Figure S4), the $\mathrm{G_b}$ values of the structures representing the adsorption of Na^+ or Cl^- ions, their co-adsorption, and the formation of NaCl solid structures in contact with the NiO facet are reported. For each surface termination, we show the most stable structure (Figure S6, top row). The Na^+ adsorption is favored only on NiO{311}+O*, whereas the co-adsorption of Na^+ an Cl^- is preferred on all the other considered terminations. The formation of locally ordered NaCl structures at the interface with NiO is not favored on any of the considered termination. The Gibbs binding energy ($\mathrm{G_b}$) of the structures involving the co-adsorption of Na^+ and Cl^- has comparable values on all the investigated O-stoichiometric NiO facets, except of NiO{100}. Indeed it is equal to -1.25 eV on NiO{111} (O), -1.08 eV on NiO{311}, and -0.99 eV on NiO{511}. As a result, the availability of the surfaces sites for the crystal growth is similar on the different NiO facets (except of NiO{100}), also because they are not covered by solid salts structures hindering the diffusion of growth units to the NiO surface. This explains the formation of spherical particles by the synthesis in molten NaCl (Figure S6b).

The surface free energies of the NiO facets at the experimental conditions of particle growth in alkali chlorides, calculated with *ab initio* thermodynamics, are reported in Figure S5. The alkali salt solid structures, which show strong Gibbs binding energies on the high-index NiO facets, reduce the energy of those facets significantly; however, the NiO{100} facet retains the lowest surface free energy at the experimental conditions, and the resulting Wulff construction does not capture the experimental particle shape. We calculated that, in order to achieve trapezohedral shapes in the Wulff construction plots, the Gibbs binding energy should be -2.0 eV for the adsorption of KCl on NiO{311}, and -2.2 eV for the adsorption of LiCl on NiO{511}. Such values are far higher in absolute value than the calculated ones (-1.10 eV for KCl and -1.12 for LiCl). Therefore, we believe that the formation of high-index particle in alkali salt media is induced by controlled growth rates from ordered salts acting as capping agents.

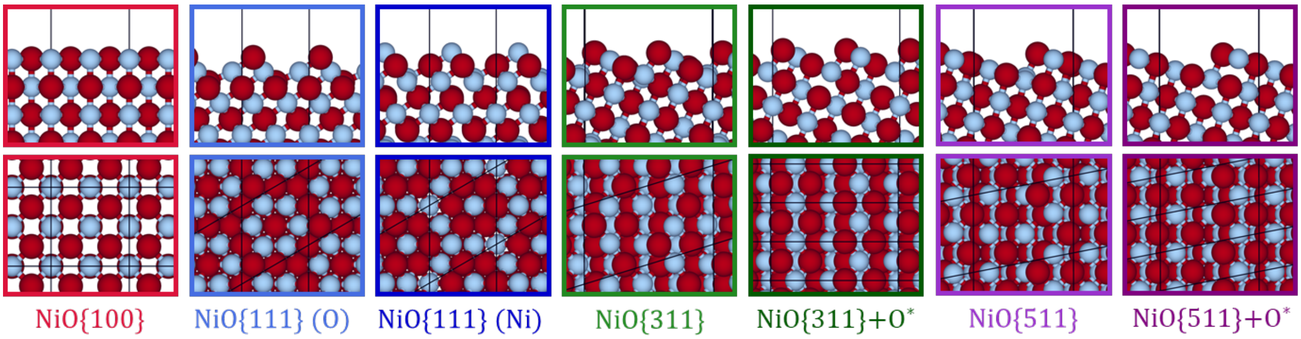

Figure S4. Top and lateral views of the NiO structures without adsorbates (except of over-stoichiometric oxygen) which are the most stable structures in the range between 0 and 1000 °C.

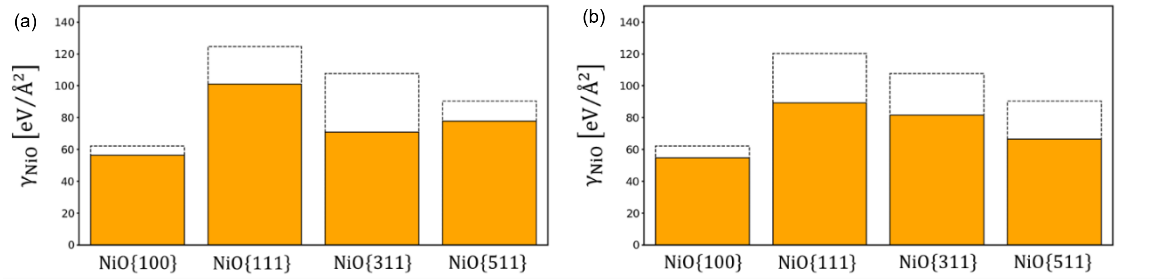

Figure S5. Surface free energy of NiO facets at 550 °C obtained with *ab initio* thermodynamics calculations in the presence of (a) KCl and (b) LiCl. Dotted lines refer to surface free energies of the clean NiO facets.

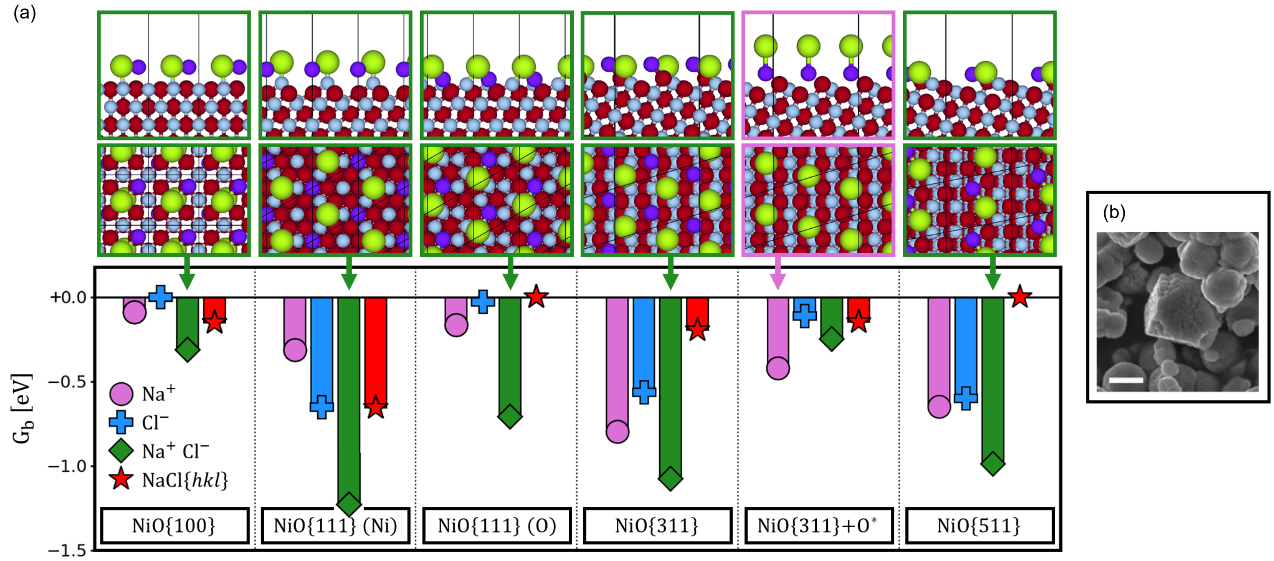

Figure S6. (a) (*Bottom*) Gibbs binding energies (per unit cell) of NiO surface structures involving the adsorption of Na^+, Cl^-, co-adsorbed Na^+ and Cl^- ions, and crystal NaCl{*hkl*} salts structures on various NiO surface terminations (indicated), calculated at 550 °C and with respect to clean NiO surfaces. (*Top*) Lateral and top views of the most strongly bound agents for each considered NiO termination (O in red, Ni in grey, Na in violet, Cl in green). (b) Experimental NiO particles produced by MSS in NaCl. Scale bar equals 1 µm.

Table S2. Gibbs binding energies (G_b) and surface free energies ($\gamma_{\{hkl\}}$) of the structures investigated upon the adsorption (and co-adsorption) of K^+, Cl^-, and extensive KCl structures on NiO facets. The salt structures that did not maintain a crystalline structure are indicated as disordered.

NiO{100}	G_b [eV]	$\gamma_{\{hkl\}}$ [meV/Å²]
clean		62.1
liquid KCl	-0.044	60.9
K^+ (0.125ML)	-0.115	58.9
K^+ (0.25ML)	-0.215	56.2
K^+ (0.50ML)	0.280	69.8
Cl^- (0.25ML)	0.120	65.4
Cl^- (0.50ML)	0.505	76.0
K^+ Cl^- (0.50ML)	-0.178	57.2
K^+ Cl^- (1.00ML)	-0.005	61.9
KCl {100}	-0.147	58.0
KCl {110}	disordered	
KCl {111}	0.919	87.4

NiO{111} (Ni)	G_b [eV]	$\gamma_{\{hkl\}}$ [meV/Å²]
clean		124.6
liquid KCl	-0.034	123.6
K^+ (0.50ML)	-0.143	120.1
Cl^- (0.50ML)	-0.516	108.3
K^+ Cl^- (1.00ML)	-0.746	101.0
KCl {100}	0.910	153.6
KCl {111}	-0.202	118.2

NiO{111} (O)	G_b [eV]	$\gamma_{\{hkl\}}$ [meV/Å²]
clean		120.2
liquid KCl	-0.015	119.8
K^+ (0.50ML)	-0.052	118.6
Cl^- (0.50ML)	0.496	136.0
K^+ Cl^- (1.00ML)	-0.184	114.4
KCl {100}	disordered	
KCl {111}	0.047	121.7

NiO{111} + 0.50ML O*	G_b [eV]	$\gamma_{\{hkl\}}$ [meV/Å²]
clean		196.9
liquid KCl	-0.034	195.8
K^+ (0.083ML)	-0.208	190.3
K^+ (0.25ML)	-0.519	180.4
K^+ (1.00ML)	0.218	203.8
KCl {100}	0.467	211.7
KCl {111}	-0.151	192.1

NiO{311}	G_b [eV]	$\gamma_{\{hkl\}}$ [meV/Å²]
clean		107.4
liquid KCl	-0.070	105.1
K^+ (0.125ML)	-0.189	101.2
K^+ (0.25ML)	-0.319	96.8
K^+ (0.50ML)	-0.351	95.8
Cl^- (0.25ML)	-0.330	96.5
Cl^- (0.50ML)	-0.145	102.6
K^+ Cl^- (0.50ML)	-0.373	95.1
K^+ Cl^- (1.00ML)	-0.781	81.5
KCl {100}	-1.101	70.9
KCl {110}	-0.594	87.7
KCl {111}	disordered	

NiO{311} + 0.50ML O*	G_b [eV]	$\gamma_{\{hkl\}}$ [meV/Å²]
clean		122.3
liquid KCl	-0.080	119.7
K^+ (0.125ML)	-0.135	117.9
K^+ (0.50ML)	-0.350	110.7
Cl^- (0.50ML)	0.670	144.6
K^+ Cl^- (1.00ML)	0.500	138.9
KCl {100}	-0.239	114.4
KCl {110}	-0.099	119.0

NiO{511}	G_b [eV]	$\gamma_{\{hkl\}}$ [meV/Å²]
clean		90.3
liquid KCl	-0.027	89.7
K^+ (0.50ML)	-0.034	89.5
Cl^- (0.50ML)	-0.194	86.1
K^+ Cl^- (1.00ML)	-0.594	77.7
KCl {310}	1.108	113.7
KCl {311}	3.829	171.4

NiO{511} + 0.50ML O*	G_b [eV]	$\gamma_{\{hkl\}}$ [meV/Å²]
clean		102.7
liquid KCl	-0.056	101.5
K^+ (0.50ML)	-0.239	97.6
Cl^- (0.50ML)	0.138	105.6
K^+ Cl^- (1.00ML)	0.050	103.7
KCl {310}	disordered	

Table S3. Gibbs binding energies (G_b) and surface free energies ($\gamma_{\{hkl\}}$) of the structures investigated upon the adsorption (and co-adsorption) of Li^+, Cl^-, and extensive LiCl structures on NiO facets. The salt structures that did not maintain a crystalline structure are indicated as disordered.

NiO{100}	G_b [eV]	$\gamma_{\{hkl\}}$ [meV/Å²]
clean		62.1
liquid LiCl	-0.072	60.1
Li^+ (0.125ML)	-0.126	58.6
Li^+ (0.25ML)	-0.222	56.0
Li^+ (0.50ML)	-0.083	59.8
Cl^- (0.25ML)	0.435	74.1
Cl^- (0.50ML)	0.745	82.6
Li^+ Cl^- (0.50ML)	-0.268	54.7
Li^+ Cl^- (1.00ML)	-0.027	61.3
LiCl {100}	0.403	73.2
LiCl {110}	disordered	
LiCl {111}	0.447	74.4

NiO{111} (Ni)	G_b [eV]	$\gamma_{\{hkl\}}$ [meV/Å²]
clean		124.6
liquid LiCl	-0.085	121.9
Li^+ (0.50ML)	-0.161	119.5
Cl^- (0.50ML)	-0.523	108.0
Li^+ Cl^- (1.00ML)	-0.899	96.1
LiCl {100}	disordered	
LiCl {111}	0.145	129.3

NiO{111} (O)	G_b [eV]	$\gamma_{\{hkl\}}$ [meV/Å²]
clean		120.2
liquid LiCl	-0.306	110.5
Li^+ (0.50ML)	-0.523	103.6
Cl^- (0.50ML)	0.385	132.5
Li^+ Cl^- (1.00ML)	-0.975	89.2
LiCl {100}	disordered	
LiCl {111}	-0.647	99.7

NiO{111} + 0.50ML O*	G_b [eV]	$\gamma_{\{hkl\}}$ [meV/Å²]
clean		196.9
liquid LiCl	-0.034	195.8
Li^+ (0.083ML)	-0.113	193.3
Li^+ (0.25ML)	-0.275	188.1
Li^+ (1.00ML)	0.293	206.2
LiCl {100}	-0.747	173.2
LiCl {111}	-0.759	172.8

NiO{311}	G_b [eV]	$\gamma_{\{hkl\}}$ [meV/Å²]
clean		107.4
liquid LiCl	-0.065	105.3
Li^+ (0.125ML)	-0.197	100.9
Li^+ (0.25ML)	-0.393	94.4
Li^+ (0.50ML)	-0.482	91.4
Cl^- (0.25ML)	-0.325	96.6
Cl^- (0.50ML)	-0.208	100.5
Li^+ Cl^- (0.50ML)	-0.374	95.0
Li^+ Cl^- (1.00ML)	-0.782	81.5
LiCl {100}	-0.481	91.5
LiCl {110}	disordered	
LiCl {111}	disordered	

NiO{311} + 0.50ML O*	G_b [eV]	$\gamma_{\{hkl\}}$ [meV/Å²]
clean		122.3
liquid LiCl	-0.080	119.7
Li^+ (0.125ML)	-0.163	116.9
Li^+ (0.50ML)	-0.356	110.5
Cl^- (0.50ML)	0.835	150.0
Li^+ Cl^- (1.00ML)	0.665	144.4
LiCl {100}	0.021	123.0
LiCl {110}	-0.069	120.0

NiO{511}	G_b [eV]	$\gamma_{\{hkl\}}$ [meV/Å²]
clean		90.3
liquid LiCl	-0.082	88.5
Li^+ (0.50ML)	-0.487	79.9
Cl^- (0.50ML)	-0.198	86.1
Li^+ Cl^- (1.00ML)	-0.759	74.2
LiCl {310}	-1.123	66.5
LiCl {311}	0.441	99.6

NiO{511} + 0.50ML O*	G_b [eV]	$\gamma_{\{hkl\}}$ [meV/Å²]
clean		102.69
liquid LiCl	-0.056	101.51
Li^+ (0.50ML)	-0.162	99.26
Cl^- (0.50ML)	0.119	105.20
Li^+ Cl^- (1.00ML)	0.051	103.76
LiCl {310}	0.557	114.48

Table S4. Gibbs binding energies (G_b) and surface free energies ($\gamma_{\{hkl\}}$) of the structures investigated upon the adsorption (and co-adsorption) of Na^+, Cl^-, and extensive NaCl structures on NiO facets.

**NiO{100}**	G_b [eV]	$\gamma_{\{hkl\}}$ [meV/Å²]
clean		62.1
Na^+ (0.25ML)	-0.089	59.6
Cl^- (0.25ML)	0.019	62.6
Na^+ Cl^- (0.50ML)	-0.313	53.5
NaCl {100}	-0.154	57.9

**NiO{111} (Ni)**	G_b [eV]	$\gamma_{\{hkl\}}$ [meV/Å²]
clean		124.6
Na^+ (0.50ML)	-0.312	114.7
Cl^- (0.50ML)	-0.652	103.9
Na^+ Cl^- (1.00ML)	-1.245	85.1
NaCl {111}	-0.658	103.7

**NiO{111} (O)**	G_b [eV]	$\gamma_{\{hkl\}}$ [meV/Å²]
clean		120.2
Na^+ (0.50ML)	-0.164	115.0
Cl^- (0.50ML)	-0.028	119.3
Na^+ Cl^- (1.00ML)	-0.708	97.7
NaCl {111}	0.491	135.8

**NiO{311}**	G_b [eV]	$\gamma_{\{hkl\}}$ [meV/Å²]
clean		107.4
Na^+ (0.50ML)	-0.797	81.0
Cl^- (0.25ML)	-0.563	88.7
Na^+ Cl^- (1.00ML)	-1.075	71.8
NaCl {100}	-0.197	100.9

**NiO{311} + 0.50ML O***	G_b [eV]	$\gamma_{\{hkl\}}$ [meV/Å²]
clean		122.3
Na^+ (0.50ML)	-0.420	108.4
Cl^- (0.25ML)	-0.114	118.6
Na^+ Cl^- (1.00ML)	-0.250	114.0
NaCl {100}	-0.049	117.4

**NiO{511}**	G_b [eV]	$\gamma_{\{hkl\}}$ [meV/Å²]
clean		90.3
Na^+ (0.50ML)	-0.649	76.5
Cl^- (0.50ML)	-0.600	77.6
Na^+ Cl^- (1.00ML)	-0.989	69.3

Table S5. Zero-point energies (ZPE), vibrational enthalpies (H_{vib}), entropies (S_{vib}), and Helmoltz free energies (F_{vib}) at 550 °C (per NiO monomer) of bulk NiO, and NiO surfaces.

**Vibrations contributions at 550 °C of bulk NiO and NiO surfaces**				
	ZPE [eV/NiO]	H_{vib} [eV/NiO]	S_{vib} [meV/K/NiO]	F_{vib} [eV/NiO]
NiO bulk	0.107	0.364	0.856	-0.349
NiO {100}	0.100	0.390	0.964	-0.413
NiO {111} (O)	0.097	0.363	0.958	-0.435
NiO {111} (Ni)	0.106	0.373	0.956	-0.423
NiO {311}	0.110	0.379	0.944	-0.407
NiO {511}	0.105	0.385	0.954	-0.410

Table S6. Zero-point energies (ZPE), and Helmoltz free energies (F_{vib}) at 550 °C of the structures representing the interfaces between NiO and the alkali salt surfaces. For each structure, vibrations are calculated for N_{NiO} NiO monomers and N_{ACl} alkali salt monomers.

Vibrations contributions at 550 °C of interface strtuctures

	N_{NiO} [-]	N_{KCl} [-]	ZPE [eV]	F_{vib} [eV]
NiO {100} + KCl {100}	4	2	0.545	-3.313
NiO {100} + KCl {111}	8	4	0.961	-6.493
NiO {111} (Ni) + KCl {100}	8	4	0.998	-6.605
NiO {111} (Ni) + KCl {111}	4	2	0.462	-3.417
NiO {111} (O) + KCl {111}	8	4	0.958	-6.576
NiO {111}+O* + KCl {100}	8	4	0.979	-6.760
NiO {111}+O* + KCl {111}	4	2	0.560	-3.446
NiO{311} + KCl {100}	6	2	0.773	-4.075
NiO{311} + KCl {110}	6	2	0.815	-4.239
NiO{311}+O* + KCl {100}	6	2	0.831	-4.197
NiO{311}+O* + KCl {110}	6	2	0.780	-4.090
NiO{511} + KCl {310}	6	4	0.793	-5.664
NiO{511} + KCl {311}	6	4	0.780	-5.825

	N_{NiO} [-]	N_{LiCl} [-]	ZPE [eV]	F_{vib} [eV]
NiO {100} + LiCl {100}	4	2	0.577	-2.856
NiO {100} + LiCl {111}	8	4	1.066	-5.666
NiO {111} (Ni) + LiCl {111}	4	2	0.524	-3.090
NiO {111} (O) + LiCl {111}	8	4	1.084	-6.212
NiO {111}+O* + LiCl {100}	8	4	1.031	-5.983
NiO {111}+O* + LiCl {111}	4	2	0.557	-3.183
NiO{311} + LiCl {100}	6	2	0.759	-4.060
NiO{311}+O* + LiCl {100}	6	2	0.869	-3.844
NiO{311}+O* + LiCl {110}	6	2	0.865	-3.742
NiO{511} + LiCl {310}	6	4	0.949	-4.858
NiO{511} + LiCl {311}	6	4	0.891	-4.980
NiO{511}+O* + LiCl {310}	6	4	0.941	-4.779

	N_{NiO} [-]	N_{NaCl} [-]	ZPE [eV]	F_{vib} [eV]
NiO {100} + NaCl {100}	4	2	0.585	-3.340
NiO {111} (Ni) + NaCl {111}	4	2	0.551	-3.493
NiO {111} (O) + NaCl {111}	4	2	0.551	-3.362
NiO{311} + NaCl {100}	6	2	0.769	-3.869
NiO{311}+O* + NaCl {100}	6	4	0.944	-5.313

4. References

[1] P. Giannozzi, S. Baroni, N. Bonini, M. Calandra, R. Car, C. Cavazzoni, D. Ceresoli, G. L. Chiarotti, M. Cococcioni, I. Dabo, A. Dal Corso, S. De Gironcoli, S. Fabris, G. Fratesi, R. Gebauer, U. Gerstmann, C. Gougoussis, A. Kokalj, M. Lazzeri, L. Martin-Samos, N. Marzari, F. Mauri, R. Mazzarello, S. Paolini, A. Pasquarello, L. Paulatto, C. Sbraccia, S. Scandolo, G. Sclauzero, A. P. Seitsonen, A. Smogunov, P. Umari, R. M. Wentzcovitch, *J. Phys. Condens. Matter* **2009**, *21*, 395502.

[2] J. P. Perdew, K. Burke, M. Ernzerhof, *Phys. Rev. Lett.* **1996**, *77*, 3865–3868.

[3] M. Cococcioni, S. De Gironcoli, *Phys. Rev. B - Condens. Matter Mater. Phys.* **2005**, *71*, 1–16.

[4] J. Hubbard, *Proc. R. Soc. London. Ser. A. Math. Phys. Sci.* **1963**, *276*, 238–257.

[5] B. Himmetoglu, A. Floris, S. De Gironcoli, M. Cococcioni, *Int. J. Quantum Chem.* **2014**, *114*, 14–49.

[6] I. Timrov, N. Marzari, M. Cococcioni, *Phys. Rev. B* **2018**, *98*, 085127.

[7] S. Grimme, J. Antony, S. Ehrlich, H. Krieg, *J. Chem. Phys.* **2010**, *132*, 154104.

[8] S. Grimme, S. Ehrlich, L. Goerigk, *J. Comput. Chem.* **2011**, *32*, 1456–1465.

[9] A. Hjorth Larsen, J. JØrgen Mortensen, J. Blomqvist, I. E. Castelli, R. Christensen, M. Dułak, J. Friis, M. N. Groves, B. Hammer, C. Hargus, E. D. Hermes, P. C. Jennings, P. Bjerre Jensen, J. Kermode, J. R. Kitchin, E. Leonhard Kolsbjerg, J. Kubal, K. Kaasbjerg, S. Lysgaard, J. Bergmann Maronsson, T. Maxson, T. Olsen, L. Pastewka, A. Peterson, C. Rostgaard, J. SchiØtz, O. Schütt, M. Strange, K. S. Thygesen, T. Vegge, L. Vilhelmsen, M. Walter, Z. Zeng, K. W. Jacobsen, *J. Phys. Condens. Matter* **2017**, *29*.

[10] O. Andreussi, I. Dabo, N. Marzari, in *J. Chem. Phys.*, American Institute Of PhysicsAIP, **2012**, p. 064102.

[11] G. Fisicaro, L. Genovese, O. Andreussi, S. Mandal, N. N. Nair, N. Marzari, S. Goedecker, *J. Chem. Theory Comput.* **2017**, *13*, 3829–3845.

[12] F. Pedregosa, G. Varoquaux, A. Gramfort, B. Thirion, O. Grisel, V. Dubourg, A. Passos, M. Brucher, M. Perrot, É. Duchesnay, *Scikit-Learn: Machine Learning in Python*, **2011**.

[13] L. Buitinck, G. Louppe, M. Blondel, F. Pedregosa, A. Mueller, O. Grisel, V. Niculae, P. Prettenhofer, A. Gramfort, J. Grobler, R. Layton, J. Vanderplas, A. Joly, B. Holt, G. Varoquaux, **2013**.

[14] M. W. Chase, *J. Phys. Chem. Ref. Data, Monogr. 9* **1998**, *4*, 1–1951.

[15] K. Reuter, *Catal. Letters* **2016**, *146*, 541–563.
